# Use of propensity score matching to reduce bias in economic evaluations in the context of Brazilian liver transplantation

**DOI:** 10.31744/einstein_journal/2025AO1329

**Published:** 2025-07-02

**Authors:** Leandro Seiti Anazawa, Giulia Osório Santana, Roseli Fernandes Rodrigues Amaral da Cunha, Lucas Reis Correia

**Affiliations:** 1 Hospital Israelita Albert Einstein São Paulo SP Brazil Hospital Israelita Albert Einstein, São Paulo, SP, Brazil.

**Keywords:** Liver transplantation, Survival rate, Cost-benefit analysis, Hospital costs, Waiting lists, Propensity score, Unified Health System, Brazil

## Abstract

**Objective:**

To estimate the incremental cost and survival of patients on the liver transplantation waiting list who were referred for transplantation.

**Methods:**

We analyzed a cohort of 883 patients who underwent liver transplantation within the Support Program for the Institutional Development of the Unified Health System in Brazil between 2010 and 2022. Median survival was assessed using the Kaplan-Meier method and parametric extrapolation of the survival curve. A bottom-up micro-costing approach was used to direct medical hospital costs. We conducted an economic evaluation comparing patients who received transplant with those who were listed to assess the potential of propensity score matching in reducing bias in this context.

**Results:**

The economic evaluation revealed a median survival of 15.54 and 1.07 years and a mean cost of US$ purchasing power parity of 162,821.96 and 39,044.79 for patients who received transplant and listed patients, respectively. A reduction in survival and an increase in mean costs for the listed patients were observed following propensity score matching.

**Conclusion:**

A direct comparison of survival and costs between the two patient groups may lead to biased estimates of the incremental survival or costs associated with referring a patient for a liver transplantation procedure. Propensity score matching reduces the bias from differences in health status between these two patient groups, enabling more accurate estimates of the incremental survival and cost associated with referring a patient for transplantation.

## In Brief

Anazawa et al. show that matching methodologies are beneficial in health economic evaluations to reduce the bias originating from sample selection. The cost-effectiveness of the liver transplantation procedure improved after sample balancing.

## Highlights

■ Matching methodologies may reduce bias in economic evaluations.

■ Liver transplantation within the Proadi-SUS context has an incremental cost-effectiveness ratio below the Health Technology Incorporation in the Brazilian Health System threshold.

■ The patients who received transplant had a median survival of 10.1 years after list enrollment.

■ The mean cost per transplanted patient was US$ PPP 162,821.96.

## Graphical abstract

**Figure:**
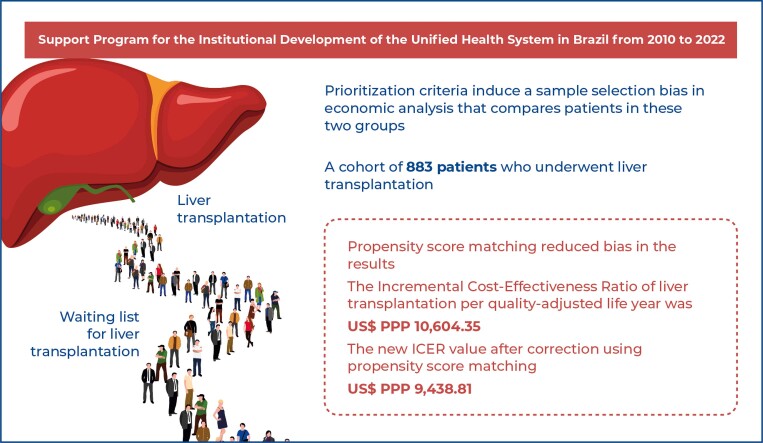

## INTRODUCTION

Economic evaluations are used in the healthcare context to compare alternative health technologies and assess the incremental costs and effectiveness of adopting new technologies.^1^ The Health Technology Incorporation in the Brazilian Health System (CONITEC - *Comissão Nacional de Incorporação de Tecnologias no Sistema Único de Saúde*) evaluates and monitors the incorporation of technologies in healthcare. These economic evaluations are conducted to support the recommendations to the Ministry of Health and play a valuable role in shaping healthcare systems. However, they also have certain shortcomings. For example, directly comparing two alternative health treatments may introduce bias into the results owing to specific contextual factors. Consequently, the results may reflect the differences between treatments as well as capture the differences related to these factors.

Matching methodologies used to establish comparable contexts can help avoid this bias, ensuring that the only difference between them is the health technology adopted. In solid organ transplantation, this approach has recently been applied to verify the influence of coronavirus disease 2019 on the outcomes of patients who underwent transplantation and those who did not.^2-4^ A matching methodology was used in the context of pre-transplant malignancy to reduce the impact of confounding factors in the sample, isolating only the effects of pre-transplant malignancy on patient survival.^5^

The liver transplantation (LT) procedure has been incorporated into Brazil’s public healthcare system—the Unified Health System (SUS - *Sistema Único de Saúde*). Approximately 95% of solid organ transplants in Brazil are financed by the government.^6^ Most public funding for transplantation procedures is provided through reimbursements for performed procedures. However, some non-profit hospitals have alternative financing methods for these procedures, particularly those recognized by the Brazilian Ministry of Health for their excellence and participation in the Support Program for the Institutional Development of the Unified Health System (Proadi-SUS - *Programa de Apoio ao Desenvolvimento Institucional do Sistema Único de Saúde*).^7^

The Ministry of Health recognizes the excellence of non-profit hospitals through ongoing and comprehensive certification processes that assess the quality of their services.^8^ Once recognized, these hospitals can apply to participate in Proadi-SUS, which provides support to the Ministry of Health and its technical areas through projects related to training, research, health technology assessment, management, and specialized care. This support is funded by the financial resources derived from tax immunity.

Given the longevity and significance of the transplantation project under analysis, assessing the outcomes at each step of the transplantation process is essential. Therefore, the present study focused on the transition between treatment on the waiting list and the transplantation procedure, as well as follow-up treatment. This evaluation is crucial because of its direct impact on patients undergoing LT at the study center. Furthermore, the project facilitates the dissemination of knowledge and techniques of solid organ transplants to other hospitals, enhancing the capacity of transplantation centers. The present study aimed to examine the potential for bias reduction in economic evaluations using propensity score matching (PSM)^9^ in the context of LT within the Proadi-SUS in Brazil.

## OBJECTIVE

To estimate the incremental cost and survival of patients on the liver transplantation waiting list who were referred for transplantation.

## METHODS

The evaluation in this study was based on the Proadi-SUS perspective. The non-profit hospital that participated in this study is an institution under the Proadi-SUS, which has been implementing the project “Support for the Management and Development of Organ and Tissue Donation, Procurement, and Transplantation in Brazil” since 2009. One of the primary objectives of this hospital is to perform LT, particularly in complex cases, such as fulminant hepatitis or hepatocellular carcinoma. The hospital adheres to the Brazilian National Transplant System guidelines and receives patients from the unified national public waiting list. Patients on the waiting list are those who are awaiting organ availability for transplantation from across Brazil. The study was reviewed and approved by the local Research Ethics Committee of the *Hospital Israelita Albert Einstein* (HIAE) and registered under the numbers CAAE: 67303022.6.0000.0071; # 5.972.008.

### Patients

A cohort of adults aged ≥18 years who were enrolled in the LT waiting list was included. Patients on the LT waiting list between January 1, 2010, and May 31, 2022, at the HIAE, a hospital in São Paulo, Brazil, within the scope of Proadi-SUS, were included in the analysis. In the sample of patients who underwent LT and were subsequently followed up (n=883), those who received transplants involving more than one solid organ or underwent retransplantation were excluded. The comparison group comprised patients who remained on the waiting list and did not receive organ transplants during the study period (n=653). Those on the waiting list received standard treatment and care based on the progression of their disease while they waited for a compatible organ for transplantation. All transplanted organs were from deceased donors, and follow-up information for patients in the study sample was obtained from the HIAE’s internal records.

### Survival analysis

The survival interval of interest in this study was defined as the period between the date of enrollment in the waiting list and the date of death. The Kaplan-Meier method was used to address the potential issue of survival data censoring.^10^ Mean and median survival rates were analyzed, and parametric extrapolations of the survival curve were employed in cases where the observed data did not allow for such estimates. Exponential, Weibull, log-logistic, log-normal, and generalized gamma distributions, as well as flexible spline methods, were used for extrapolations.^11^ The criterion for selecting the best extrapolation method involved identifying the extrapolation that best fit the observed survival curve and yielded the smallest value in the Akaike information criterion (AIC) test.^11,12^Utility estimates obtained from the literature were used in the cost-utility analysis. The estimated utility was 0.80 and 0.71 for patients who received transplant and listed patients, respectively.^13-15^

### Costs

Cost information was obtained from the HIAE’s financial records using data from the inputs and procedures associated with the medical records of the patients that were analyzed. The bottom-up micro-costing approach to direct medical and hospital costs was employed, aligning with the Proadi-SUS perspective. The cost information in medical records included the allocated use of inputs and procedures among patients, enabling the calculation of the nominal and daily aggregated values of these inputs and procedures for each patient. The costs of medications and procedure-related items were based on the average acquisition values, whereas the average charges from the responsible department within the hospital were used to determine the costs of examinations, healthcare professional teams, and hospital infrastructure usage.

The cost information included all inputs and procedures from the moment the patients were enrolled on the waiting list to the date of last follow-up or death. The costs of immunosuppressants in post-transplant treatment were not considered, as they are covered by the SUS and not captured within the scope of Proadi-SUS costs.

Owing to the presence of censored data in the present study, the Kaplan-Meier sample-average (KMSA) methodology was used to mitigate potential bias in the analyzed costs.^16,17^ Kaplan-Meier sample-average was performed using monthly intervals, and the values were calculated using the present value with a discount rate of 5% per year, as mandated by the Brazilian Ministry of Health.^1^ All costs were adjusted using the Extended National Consumer Price Index of the Brazilian Institute of Geography and Statistics for December 2021 and were converted to US$ PPP using the purchasing power parities of the Organization for Economic Co-operation and Development for the same period.

### Propensity score matching and economic evaluation

A comparison was performed between patients on the waiting list and those referred for transplantation using the incremental cost-effectiveness ratio (ICER). Incremental cost-effectiveness ratio represents the incremental cost of 1 year of survival when referring a patient for the transplantation instead of keeping them on the waiting list. Probabilistic sensitivity analyses of factors affecting the ICER were also conducted to investigate the uncertainty surrounding the parameter estimates obtained. A stratified nonparametric bootstrap procedure was used, with 1,000 random samplings with replacement. Patients were stratified into transplanted and listed groups.

Patients who remain on the waiting list are not the most appropriate comparison group for those who received transplants due to the functioning of the LT waiting list and its prioritization criteria. Consequently, we performed PSM to establish comparable groups.^5^ Propensity score matching is used to estimate the probability that each patient in the sample will receive a transplant, conditioned on factors related to potential differences in costs and patient survival observed during the listing period.^18^ If the PSM methodology works effectively in the analyzed context, patients from both groups are expected to show similarities in these factors after the matching analysis. Additionally, among the listed patients, an increase in costs and a reduction in survival are expected owing to the balancing process, where patients similar to the transplanted group are selected, particularly those with worse health conditions and increased health expenses.

Propensity score matching was conducted using nearest-neighbor matching with replacement. A ratio of one listed patient to each transplanted patient was used. After matching, survival and cost estimates were calculated solely for the matched sample. The propensity score was estimated using a logistic regression model, with observed variables, such as the Model for End-Stage Liver Disease (MELD) score at the time of enrollment, presence of acute or subacute liver failure during the listing period and prior to transplantation, sex of the patient, blood type, and year of enrollment on the list, used as controls to estimate the propensity score.

## RESULTS

### Patients

At the time of enrollment, the mean age of patients who received transplant and listed patients was 53.12 and 54.59 years, respectively (*t*-test, p=0.016; Table 1). The proportion of women was lower in the transplant group than among the listed patients (27.5% *versus* 33.8%, χ^2^ test, p=0.009; Table 1).

**Table 1:** Descriptive statistics of patients who received transplant and listed patients for a liver transplant within the Proadi-SUS context

	Transplanted (n=883)		Listed (n=653)		Difference of mean test
	Mean	Standard deviation	Mean	Standard deviation	p value
Age on enrollment in the list	53.12	12.03	54.59	11.77	0.016
Proportion of women	0.275	0.447	0.338	0.474	0.009
MELD score on enrollment in the list	28.88	5.68	22.55	10.01	0.000
Proportion of patients with acute or subacute liver failure in the list	0.026	0.159	0.011	0.103	0.050
Proportion of patients with blood type A	0.385	0.487	0.389	0.488	0.918
Proportion of patients with blood type B	0.154	0.361	0.069	0.253	0.000
Proportion of patients with blood type O	0.417	0.493	0.512	0.500	0.000
Proportion of patients with blood type AB	0.044	0.206	0.031	0.172	0.218

*t-*test for differences in mean age and MELD score. χ^2^ test for the other variables.

MELD: Model for End-stage Liver Disease.

Regarding the risk of mortality, at the time of enrollment, patients who received transplant and listed patients had a mean MELD score of 28.88 and 22.55, respectively (*t*-test, p=0.000; Table 1). Notably, the MELD score was obtained at the time of enrollment to ensure a comparable context between transplanted and listed patients, reflecting the moment both groups were enrolled. Therefore, these values indicate that patients referred for transplantation were enrolled in the waiting list with higher MELD scores, reflecting a higher risk of mortality.

A higher proportion of patients with acute or subacute liver failure was observed at the time of listing (2.6% *versus* 1.1%, χ^2^ test, p=0.050). Among patients who received transplant, the proportion of patients with blood type B was higher compared to the listed patients (15.4% *versus* 6.9%, χ^2^ test, p=0.000). Meanwhile, among the listed patients, the proportion of patients with blood type O was higher compared to the patients who received transplant (51.2% *versus* 41.7%, χ^2^test, p=0.000; Table 1).

### Survival

**Figure 1:**
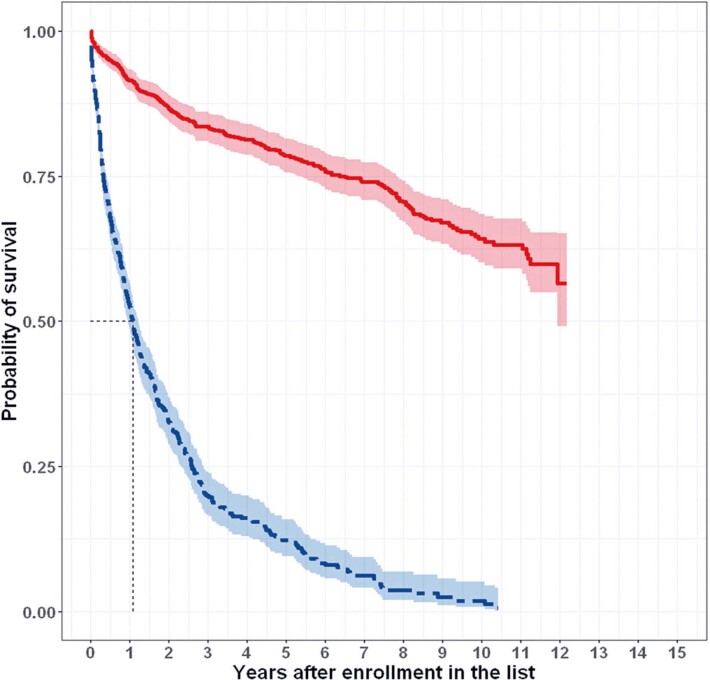
Survival curves of patients who received transplant (solid and red curve) and listed patients for liver transplant (dashed and blue curve)

Patient survival, estimated using the Kaplan-Meier method, showed distinct patterns for the two patient groups (transplanted and listed), as illustrated in figure 1. The survival curve for patients who received transplant (Figure 1) indicated a survival rate of 91.4%, 78.5%, and 64.1% in the first, fifth, and tenth year after enrollment in the waiting list. In contrast, a more pronounced decline was observed in the survival curve of the listed patients (Figure 1), with survival rates of 52.4%, 12.2%, and 1.8% in the first, fifth, and tenth year after enrollment. These data indicate that patients listed for LT had significantly lower survival rates compared to those who received a transplant (Mantel-Cox test, p=0.000).

Kaplan–Meier survival curves. Data from patients enrolled in the transplant list between January 1, 2010, and May 31, 2022, at *Hospital Israelita Albert Einstein*, São Paulo City, Brazil, were within the scope of Proadi-SUS. Mantel-Cox test, p = 0.000. The shaded areas of the survival curves indicate 95% confidence intervals.

Figure 1 shows that the survival curve for patients who received transplant did not drop below a 50% probability of survival, resulting in high uncertainty regarding the shape of the remaining curve and the estimation of mean survival. The mean survival was extrapolated from both survival curves, as neither curve reached a 0% probability of survival. Parametric extrapolations of these curves were performed using exponential, Weibull, log-logistic, log-normal, generalized gamma, and flexible spline distributions. The best fit to the observed survival curve for the patients who received transplant was obtained using extrapolations of flexible spline models with 2 and 3 nodes. Additionally, the 3-node spline extrapolation exhibited the lowest AIC value (1885.3). Therefore, a 3-node spline was used to extrapolate the survival curve of the transplant group and estimate the mean survival for this group. For the listed patients, the same methodological process resulted in the selection of the Weibull distribution for extrapolation.

The mean survival estimate for the transplant and listed groups was 21.59 and 2.04 years, respectively. However, the mean survival estimate for the transplant group was uncertain because of the lack of complete information on the survival curve. Consequently, we used the median survival estimates in subsequent analyses, as they are associated with a lower degree of error. Median survival estimates of 15.54 and 1.07 years were obtained from the extrapolated survival curve for the transplant group and the observed survival curve for the listed group, respectively. As survival was calculated from the same time point (enrollment in the list) for both groups, the results indicated that referring a patient for transplantation may result in a median survival benefit of 14.47 years.

### Costs

Based on the Proadi-SUS perspective, the costs included all expenses related to the patients in the sample, which were allocated to the project within the scope of the Proadi-SUS at *Hospital Israelita Albert Einstein*. The observed aggregated costs from the patients in the sample indicated that the categories of medication, examinations, health professional staff, and materials represented the majority of the costs for both patient groups (Table 2).

**Table 2:** Observed mean cost (US$ PPP) of the analyzed procedures by patient group for a liver transplant within the Proadi-SUS context

Observed cost (US$ PPP) per macro category from January 1, 2010, to May 31, 2022				
	Transplanted (n=883)		Listed (n=653)	
	Total cost	Cost/patient ratio	Total cost	Cost/patient ratio
Medication	100,379,505.15	113,680.07	18,545,406.44	28,400.32
Examinations	11,183,944.28	12,655.85	1,664,855.38	2,549.55
Health professional staff care	8,463,609.62	9,585.06	1,477,696.62	2,262.94
Materials	5,352,074.67	6,061.24	265,842.80	407.11
Other items	114,565.13	129.75	27,143.29	41.57
**Observed mean cost (US$ PPP) per transplantation phase from January 1, 2010, to May 31, 2022**				
	**Transplanted (n=883)**	**Listed (n=653)**		
Pre-transplant phase	30,560.22	12,796.00		
Surgery phase	58,852.56			
Post-transplant phase	59,377.58			

Total cost and number of patients between January 1, 2010, and May 31, 2022. Data were obtained from the *Hospital Israelita Albert Einstein*. Medication, examinations, and material costs include all items used throughout the patient’s treatment, excluding post-transplant immunosuppressant medication costs for patients in the transplant group. Health professional staff care includes the costs associated with receiving medical, nursing, and anesthesia professional care. Other items include the costs associated with taxes, hospital ward usage, and other facilities. The values deflated by the general IPCA based on December 2021 were converted to US$ PPP 2021.

IPCA: Extended National Consumer Price Index (Índice Nacional de Preços ao Consumidor Amplo); PPP: purchasing power parity; US$: U.S. dollar.

The observed mean costs for those who received transplants were higher in the transplant surgery and post-transplant phases, amounting to US$ PPP 58,852.56 and US$ PPP 59,377.58, respectively (Table 2). In the pre-transplantation phase, the mean cost for these patients was US$ PPP 30,560.22, whereas it was US$ 12,796.00 for the listed patients. After applying the KMSA, the mean total cost for patients who received transplant and listed patients was US$ PPP 162,821.96 and 39,044.79, respectively (Table 3).

**Table 3:** Incremental cost-effectiveness ratio (in US$ PPP of December 2021) for referring a patient for liver transplant within the context of Proadi-SUS, before and after the propensity score matching

	Number of observations	Survival (median years)	Mean cost per patient (US$ PPP)	Survival difference (years)	Cost difference (US$ PPP)	ICER (US$ PPP)	ICER/QALY (US$ PPP)
Before PSM							
Transplanted	883	15.54	162,821.96	14.47	123,777.17	8,554.05	10,604.35
Listed	653	1.07	39,044.79				
After PSM							
Transplanted	883	15.54	162,821.96	14.71	111,781.00	7,598.98	9,438.81
Listed	246	0.83	51,040.96				

ICER: incremental cost-effectiveness ratio; PPP: purchasing power parity; PSM: propensity score matching; QALY: quality-adjusted life years; US$: U.S. dollars.

### Propensity score matching and economic evaluation

**Figure 2:**
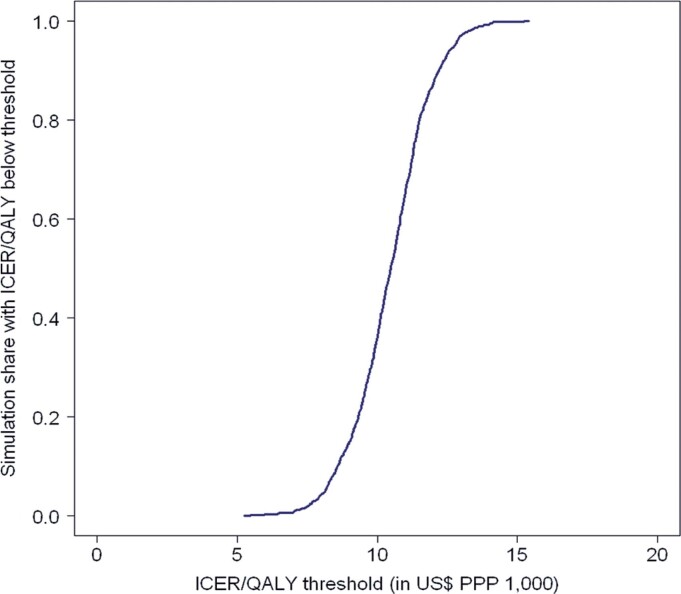
Cost-effectiveness acceptability curve based on 1,000 non-parametric bootstrap simulations

ICER: incremental cost-effectiveness ratio; PPP: purchasing power parity; QALY: quality-adjusted life years; US$: U.S. dollars.

Table 3 presents the cost-effectiveness analysis (CEA) of LT compared with treatment for those on the waiting list for LT, before and after PSM correction. Before PSM correction, LT was associated with a higher median survival (15.54 years) and a higher mean cost per patient (US$ PPP 162,821.96) than those on the waiting list for LT, who had a median survival of 1.07 years and cost of US$ 39,044.79. Referring the patient for LT resulted in an ICER of US$ PPP 8,554.17 per year of life gained or US$ PPP 10,604.35 per quality-adjusted life year (QALY). Furthermore, probabilistic sensitivity analysis revealed that 100% of the simulations exhibited an ICER below the threshold proposed by CONITEC (PPP 16,275.20) for decisions regarding the incorporation of new technologies into the SUS (Figure 2).^19^

Following PSM, the survival of patients who received transplant did not change, whereas the survival of listed patients decreased (from 1.07 to 0.83 years). Additionally, the average cost per listed patient increased from US$ PPP 39,044.79 to US$ PPP 51,040.96 (Table 3). This indicates that, in the matching analysis, listed patients with higher healthcare costs were retained more often than those referred for LT, reflecting the tendency to patients with worsening health conditions and higher costs. The ICER per QALY decreased from US$ PPP 10,604.35 to 9,438.81 (Table 3), indicating that when analyzing patients with similar characteristics affecting survival and costs, LT within the scope of Proadi-SUS was even more cost-effective than in the uncorrected observational data context.

The result of the PSM methodology was robust in balancing the two patient groups, successfully eliminating most differences in the observed characteristics considered in the analysis (Table 4). Among the 20 variables used to balance the groups, four variables (blood type B, blood type O, year of enrollment in the list - 2010, and year of enrollment in the list - 2019) still exhibited statistically significant differences in mean values following PSM. However, PSM effectively balanced variables associated with the patient’s health status, such as the MELD score at enrollment and the presence of acute or subacute LF at the time of listing. This balance was achieved by restricting the analyzed sample, resulting in the exclusion of 407 listed patients.

**Table 4:** Descriptive statistics of patients who received transplant and listed patients for a liver transplant within the context of Proadi-SUS, before and after propensity score matching at a statistical significance of 5% in the mean difference test

	Before balancing		p value	After balancing		p value
	Transplant group (mean)	Listed patients (mean)		Transplant group (mean)	Listed patients (mean)	
MELD score on at the time of listing	28.88	22.55	0.000	28.88	29.70	0.065
Acute or subacute liver failure at the time of listing	0.03	0.01	0.032	0.03	0.02	0.610
Women	0.28	0.34	0.008	0.28	0.32	0.158
Blood type A	0.39	0.39	0.876	0.39	0.37	0.883
Blood type B	0.15	0.07	0.000	0.15	0.12	0.027
Blood type O	0.42	0.51	0.000	0.42	0.47	0.010
Blood type AB	0.04	0.03	0.172	0.04	0.04	0.161
Year of enrollment in the list – 2010	0.13	0.07	0.000	0.13	0.09	0.021
Year of enrollment in the list – 2011	0.13	0.09	0.018	0.13	0.13	0.182
Year of enrollment in the list – 2012	0.10	0.09	0.569	0.10	0.09	0.568
Year of enrollment in the list – 2013	0.10	0.12	0.254	0.10	0.09	0.644
Year of enrollment in the list – 2014	0.09	0.11	0.342	0.09	0.09	0.866
Year of enrollment in the list – 2015	0.08	0.09	0.504	0.08	0.09	0.474
Year of enrollment in the list – 2016	0.06	0.08	0.128	0.06	0.06	0.805
Year of enrollment in the list – 2017	0.05	0.03	0.055	0.05	0.04	0.548
Year of enrollment in the list – 2018	0.06	0.06	0.752	0.06	0.07	0.903
Year of enrollment in the list – 2019	0.06	0.09	0.010	0.06	0.08	0.032
Year of enrollment in the list – 2020	0.06	0.05	0.352	0.06	0.07	0.464
Year of enrollment in the list – 2021	0.08	0.09	0.252	0.08	0.09	0.825
Year of enrollment in the list – 2022	0.00	0.02	0.000	0.00	0.01	0.628

Two-sample *t*-test for difference in mean values.

MELD: Model for End-stage Liver Disease.

## DISCUSSION

Matching methodologies are beneficial during economic evaluations in cases of solid organ transplantation, specifically when inherent processes may influence the costs and survival of the patients analyzed beyond the treatment. Following PSM, the survival of listed patients decreased (from 1.07 to 0.83 years), and the average cost per listed patient increased from US$ PPP 39,044.79 to US$ PPP 51,040.96. These results indicate successful reduction of bias in patient selection for LT within the Proadi-SUS context using the PSM. The ICER for referring patients for transplantation after PSM, even in the complex cases of LT procedures, including those involving fulminant hepatitis or hepatocellular carcinoma, was US$ 9,438.81. This value is below the ICER threshold proposed by CONITEC, indicating that this procedure is cost-effective in the present context.^19^

The median survival estimates for patients who received LT were 15.54 years, compared with 1.07 years for those who remained on the list. This result indicates that referring patients for LT increases their median survival by 14.47 years, along with improving their quality of life. Additionally, the estimated median survival of patients who underwent transplantation in the analyzed sample was within the range of 10-20 years reported in the literature.^20-25^ For listed patients, the median survival estimated in the literature ranges from 1.9 to 4 years.^12,14,20,24^ Although the mean estimate is more commonly used in economic evaluations, we used the median estimate because of its lower level of uncertainty and lower degree of error in the analyzed context.

Only one study has used a similar methodology and comparison groups, yielding comparable results, which estimated the median survival of patients in the UNOS database between 1987 and 2012.^20^ This study reported a median survival of 10.1 and 2.9 years for the transplant and listed groups, respectively. The differences in results can be primarily attributed to variations in the time period, transplantation techniques, and organizational structures between UNOS and the analyzed contexts.

Before applying the KMSA, the mean cost per patient in the pre-transplantation phase was US$ PPP 30,560.22 for patients who underwent transplantation and US$ PPP 12,796.00 for those who remained on the list. In contrast, the mean costs per patient in the transplant and post-transplant phases were US$ PPP 58,852.56 and 59,377.58, respectively. The values for the pre-transplant phase were higher for patients in the transplant group and lower for those who remained on the list compared to the pre-transplant costs ranging from US$ PPP 19,189.64 to 19,705.52 reported in Brazilian literature.^26,27^ The values for the transplant phase were within the range indicated in the Brazilian literature, between US$ PPP 29,148.37 and 63,567.89.^26-29^ However, comparing cost values across different studies may not yield accurate results and requires careful consideration of the patients’ health status, treatment timelines, and the cost correction methodologies used in the analyses.

Cost-effectiveness analysis conducted in this study indicated that referring a patient for a single LT exhibited an ICER of US$ PPP 10,604.35 per QALY compared with keeping the patient on the waiting list for LT. This ICER is below the threshold (US$ PPP 16,275.20) proposed by CONITEC for incorporating new health technologies into the Brazilian public health system. When considering the threshold for severe diseases (US$ PPP 48,825.60), the ICER for the analyzed LT was even lower, suggesting that LT is a cost-effective procedure when evaluating the incorporation of a new technology into the SUS. However, considering other criteria, including budgetary impact, patient preferences, and socioeconomic and ethical aspects, is important when evaluating health technologies. Additionally, as LT is already incorporated into the Brazilian public health system, this procedure remained cost-effective, even in cases of highly complex LT procedures. Probabilistic sensitivity analysis confirmed the robustness of results.

The process of selecting and prioritizing patients for LT resulted in transplant and listed groups with differing characteristics that affected their survival and costs. Patients with worsening health conditions tend to be prioritized for transplantation, whereas those remaining on the waiting list generally have less severe or more stable health conditions. Comparable patient groups were established using the PSM. Consequently, ICER per QALY for the LT procedure decreased from US$ PPP 10,604.35 to 9,438.81, indicating increased cost-effectiveness. This decrease in ICER was attributed to an increase in costs for listed patients (from US$ PPP 39,044.79 to 51,040.96) and a reduction in their survival (from 1.07 to 0.83 years). This pattern suggests that PSM effectively retained only those listed patients with characteristics similar to those who received transplant. Notably, PSM matches patients only in terms of observable factors, and unobservable factors can still introduce bias into the results.

This study also has certain limitations. First, it relied on censored data, as the outcome of interest was not observed for a portion of our sample. However, we applied methodologies (Kaplan-Meier and KMSA) to address this limitation. Second, this study was conducted in a non-profit hospital setting, which limits the generalizability of our findings to other hospitals in Brazil. Although analyzed patients were drawn from national and unified transplantation waiting lists, our results are specific to the studied context and have limited external validity. Nevertheless, our study involved a large sample size (n=883) and long time span (January 2010 to May 2022), reducing the potential for bias due to the sample size.

## CONCLUSION

Our results show that matching methodologies, such as propensity score matching, are beneficial in health economic evaluations for reducing bias from sample selection. In our analysis, sample balancing the liver transplantation procedure was more cost-effective after sample balancing. Considering that another objective of the transplantation project is to disseminate knowledge to other hospitals and train their staff in solid organ transplantation, the potential impact extends beyond the directly benefited patients. These findings align with the objectives of the Proadi-SUS to continuously enhance health services for the Brazilian public health system and its population.
